# Implementation of Clinical Algorithms in Complex Wound Care: A Pilot Study on Feasibility and Professional Acceptance

**DOI:** 10.1155/nrp/1833733

**Published:** 2026-05-15

**Authors:** Marques R., Lopes M., Pais-Vieira C., Neves-Amado J., Alves P.

**Affiliations:** ^1^ Centre for Interdisciplinary Research in Health, Faculdade de Ciências da Saúde e Enfermagem, Universidade Católica Portuguesa, Porto, Portugal, ucp.pt; ^2^ School of Nursing Department, Faculdade de Ciências da Saúde e Enfermagem, Universidade Católica Portuguesa, Porto, Portugal, ucp.pt; ^3^ School of Nursing Department, Universidade Federal Ceará, Fortaleza, Brazil

**Keywords:** clinical decision support, complex wound care, feasibility study, mobile health (mHealth), nursing informatics, professional acceptance, wound assessment

## Abstract

**Purpose:**

Mobile applications that integrate clinical algorithms have the potential to standardize wound assessment and to support clinical decision‐making across assessment, diagnostic support, and therapeutic planning in complex wound care. This pilot study explored the feasibility of implementing such algorithms in a mobile application and examined concordance with nurse assessments and professional acceptance.

**Methods:**

A prospective multicenter pilot cohort study was conducted between June and October 2023 in rehabilitation units, outpatient clinics, primary healthcare, homecare, and hospitals. Adults aged ≥ 18 years with pressure injuries/pressure ulcers, venous leg ulcers, arterial ulcers, or diabetic foot ulcers were eligible. Nurses recorded data using the *CWS_Validation* application at three time points over 4 weeks. Agreement between algorithm‐generated classifications and nurse assessments was analyzed using Cohen’s kappa, Fleiss’ kappa, and Krippendorff’s alpha. Nurse adherence to therapeutic recommendations and perceptions of the alert system were also evaluated.

**Results:**

A total of 49 patients with 56 wounds (mean age = 79 years) were included. Pressure injuries/pressure ulcers were most prevalent (71.4%), particularly categories 3 (25%) and 4 (23.2%). The algorithm provided diagnoses for 46 wounds, with fair overall concordance with nurse assessments (*κ* = 0.41; 95% CI: 0.24–0.57). Adherence to therapeutic recommendations was high: 85.3% in complete monitoring and 90.3% in partial monitoring. Nurses rated the alert system positively (mean = 4.34, SD = 0.82) in complete monitoring sessions (*n* = 109).

**Conclusions:**

Clinical algorithms can be feasibly integrated into mobile applications and are well accepted by nurses across diverse care contexts. These preliminary findings highlight the potential of algorithm‐driven tools to support wound assessment and decision‐making, while underscoring the need for larger studies with more robust designs to confirm clinical impact.

## 1. Introduction

Chronic and complex wounds, including pressure injury/pressure ulcer (PI/PU), leg ulcers (LUs), and diabetic foot ulcers (DFUs), represent a major and growing public health concern. These wounds are associated with prolonged healing trajectories, recurrent complications, and significant impacts on morbidity, mortality, and quality of life [1–3]. They also impose substantial economic burdens on healthcare systems due to the need for long‐term treatments, repeated hospitalizations, and specialized resources [4, 5]. Optimizing wound care is therefore an international priority, particularly for vulnerable populations such as older adults and people with diabetes [6].

Evidence highlights the importance of systematic monitoring and early intervention to improve outcomes in wound management [7, 8]. However, traditional approaches remain reliant on manual documentation and subjective assessment, leading to variability between professionals and challenges in ensuring continuity and accuracy of care. In this context, digital health technologies, including mobile health (mHealth) applications, emerge as promising tools to standardize wound assessment, support decision‐making, and facilitate real‐time information exchange among healthcare providers [9, 10].

Clinical algorithms, when integrated into mHealth platforms, offer structured, evidence‐informed guidance by combining individual patient characteristics with standardized decision pathways [11]. These clinical decision support systems (CDSS) have shown potential to reduce practice variation, improve adherence to best practices, and enhance clinical documentation [12, 13]. Despite technological advances, the evidence base for algorithm‐based wound care applications in real‐world clinical practice remains relatively small and largely descriptive. Existing studies suggest promising feasibility and healthcare professionals’ acceptance, but further research is needed to establish their clinical effectiveness and agreement with clinician assessment across wound characteristics [9, 14].

This study addresses this gap by exploring the feasibility of implementing clinical algorithms for wound assessment and therapeutic decision support through a mobile application (app). Specifically, it examines (i) concordance between algorithm‐generated classifications and nurse assessments and (ii) professional acceptance, including adherence to algorithmic recommendations and perceptions of the alert system. By situating this work within routine clinical contexts, the study provides preliminary evidence to inform future large‐scale evaluations of algorithm‐driven decision support in complex wound care.

## 2. Materials and Methods

### 2.1. Design

A prospective, multicenter, observational pilot cohort study was conducted to evaluate the feasibility of implementing clinical algorithms for complex wound management in real‐world healthcare settings. Reporting was guided by the Strengthening the Reporting of Observational Studies in Epidemiology (STROBE) checklist to ensure methodological transparency and rigor [15]. In accordance with recommendations for CDSS research, external evaluation across diverse clinical contexts was prioritized to enhance applicability and reduce implementation bias [16, 17]. The multicenter design further strengthened external validity by capturing variation in wound care practices across different healthcare environments, as recommended in eHealth implementation research [18, 19].

### 2.2. Sample and Setting

Participants were consecutively recruited between June and October 2023 from rehabilitation units, outpatient clinics, primary healthcare centers, nursing homes, home care services, and hospitals across seven health institutions in urban and rural settings in northern mainland Portugal. The study included two groups:

1. Adults with complex wounds: Eligible patients were aged ≥ 18 years and presented with PI/PU (categories 1–4, unstageable, or suspected deep tissue injury), device‐related PI/PU, mucosal membrane ulcers, venous leg ulcers (VLUs), arterial ulcers, mixed ulcers, ulcers of unknown etiology, or DFU (neuropathic, ischemic, or neuroischemic). Exclusion criteria included circumferential wounds (due to technical limitations in image capture), wounds outside the specified typologies, and refusal to provide informed consent.
2. Collaborating nurses: Nurses with prior experience in wound assessment and treatment, with or without formal specialization in tissue viability, were included to ensure proper use of the application. Eligible participants were required to have at least 2 years of professional experience, to be employed at the institution, and to provide daily care to persons with wounds. Participants were selected by their respective institutions.

In total, 49 patients with 56 wounds were enrolled and monitored by 48 trained nurses.

### 2.3. Variables and Measures

The CWS_Validation app, developed for iOS and Android platforms, is a mobile decision‐support tool for nurses, structured around the nursing process. It guides a comprehensive assessment of the person and the wound, supports the diagnosis of impaired skin integrity in PI/PU, LU, and DFU, and, based on healing potential, directs nurses toward expected outcomes. It enables complete or partial monitoring with image capture, automatically generating wound measures such as length, width, and area. The app also provides general and wound‐specific recommendations, including alerts, and works online due to AI‐based image algorithms. It is designed for point‐of‐care use and ensures continuity of care through secure in‐app data storage without retaining data on the mobile device. Future development will aim to enable interoperability with institutional information systems.

Data were collected using the CWS_Validation app, including the following:

• Patient characteristics: age, sex, medical history, comorbidities (e.g., diabetes and vascular disease), medication, allergies, smoking, body mass index, and skin phototype according to the Fitzpatrick scale [20].
• Wound‐related measures: typology, anatomical location, duration, recurrence, wound edges, perilesional and surrounding skin, exudate, pain (intensity, type, and frequency), systemic/local infection signs, and vascular assessment (palpation, Doppler, ankle‐brachial index [ABI], and toe‐brachial index [TBI]).
• Monitoring type:
  ◦ *Complete monitoring* included full clinical data, image capture, and wound characterization.
  ◦ *Partial monitoring* was restricted to four local parameters (necrotic/slough tissue, infection, exudate, and maceration of wound edges/perilesional skin).
• Algorithm outputs: wound typology classification, therapeutic plan recommendations, and alerts.
• Professional acceptance: nurse agreement with therapeutic recommendations (yes/no), perceived usefulness of alerts (5‐point scale), and time spent on monitoring.

The app provides both general and specific recommendations for the therapeutic plan. General recommendations are based on wound typology; for example, in PI/PU, they include risk assessment, regular skin inspection, and adequacy of support surfaces, among others. Specific recommendations are tailored to local wound characteristics and are based on the concept of wound bed preparation [21–23].

The specific groups of variables and data fields collected by the application are summarized in Table 1.

**TABLE 1:** Groups of information and data fields collected by the CWS_Validation application.

Information group	Data fields
Patient information (complex wound)	Age, sex, relevant medical history, episode type (consultation, hospitalization), confirmation that the participant has read and signed the informed consent, skin phototype, presence of any associated disease or condition, history of previous vascular surgery, tobacco use in the last 6 months, current medication, allergies, and skin sensitivities. Absence or likelihood of no nutritional intake for more than 5 days, weight (kg), and height (m)
Wound‐related symptoms in the last 24 hours	Pain with dressing and/or debridement, pain between dressing and/or debridement, drainage or exudate, odor, itching, bleeding, cosmetic or aesthetic concern and/or distress, edema and/or swelling around wound, bulk or mass effect from wound, bulk or mass effect from dressing
Anatomical location of the wound	Identification of the wound’s anatomical location using a human body image. Based on the selected anatomical location: If the wound is on a bony prominence, mucosa, or involves a medical device: Exposed to friction, pressure, or shear forces, partial or total immobility, incontinence/moisture, associated with a medical device, mucosal involvement, limited to a single location and/or has a circular/regular shape, none of the above if the wound is on the lower limbs: edema, hyperpigmentation, white atrophy, telangiectasias, varicose veins, or varicosities, venous eczema, calluses, foot and/or toe deformities, decreased or absent sensation, absence of hair growth, foot or toe gangrene, dry necrosis, intermittent claudication, neuropathic pain, nociceptive pain, rest pain especially at night, relieved when the foot is dependent, previous amputations, bilateral presence of posterior tibial and dorsalis pedis pulses, ABI, audible biphasic or triphasic doppler sound bilaterally, TBI
Healing potential	Signs of poor local blood perfusion, possibility of correcting the wound’s underlying cause, adherence to the therapeutic regimen, availability of healthcare resources (materials and professionals) to meet the patient’s needs
Wound assessment	Estimated duration of wound existence (in months), recurrent wound, wound typology, image capture of dressing, image capture of the cleaned wound, pain (characterize intensity, frequency, and type), signs and symptoms of systemic infection presumably associated with the wound, evaluation of local infection signs and symptoms, affected tissues, topographic changes, wound dimensions, automatic feedback on wound contours calculated by image processing algorithms, tissue types in the wound bed (necrotic + slough + granulation = 100%), wound edges, perilesional skin, surrounding skin, exudate
Agreement	Opinion on automatic wound bed contouring, agreement with wound measurements (length, width, and area) presented, agreement with the percentages of tissue types presented, usefulness of the recommended therapeutic plan, and usefulness of alerts during and after the process
Specific recommendations	Description of the treatment plan to be implemented: cleaning, debridement, therapeutic options, dressing fixation, and complementary and adjunctive therapies; treatment frequency; duration of monitoring period

Abbreviations: ABI = ankle‐brachial index, TBI = toe‐brachial index.

### 2.4. Procedures

Nurses used their personal smartphones to install and access the app. Patients were monitored over a 4‐week period, with mandatory assessments at baseline, Week 2, and Week 4 (complete monitoring). Additional assessments using the partial monitoring protocol could be performed as clinically indicated.

Image capture followed standardized procedures: wounds were photographed after cleaning, at a 90° angle, including at least 4 cm of perilesional skin, under controlled lighting conditions, and without flash. All data were anonymized within the app and automatically transferred to secure servers.

While using the app, nurses entered clinical data directly into the platform. After submission, the app provided real‐time feedback regarding therapeutic plans and alert indicators (e.g., signs of infection, wound size, tissues in the wound bed, and factors that delay healing). However, the app did not provide feedback on wound type during data collection. The comparison between the app‐generated wound classification and the nurses’ clinical assessments was conducted internally, ensuring blinding to prevent bias.

At the end of the data collection period, a paper‐based questionnaire containing both open‐ and closed‐ended questions was administered to assess participants’ professional profile, prior technological experience, app use, perceived usefulness, usability, barriers to adoption, perceived benefits and limitations, improvement needs, and patient and professional acceptance of the app. Overall system usability was further evaluated using the version of the System Usability Scale (SUS) validated for Portugal [24].

### 2.5. Ethical Considerations

The study was approved by the ethics committees of the Administração Regional de Saúde do Norte (Opinion No. CE/2023/58) and Unidade Local de Saúde da Guarda (Decision No. 55/2023). Written informed consent was obtained from all participants or their legal representatives. Data confidentiality and compliance with the General Data Protection Regulation (GDPR) were ensured. Participation was voluntary, without financial incentives.

### 2.6. Bias

To reduce potential sources of bias and ensure standardization, engagement, and rigor in the collection of textual and image data, training was provided to all 48 collaborating nurses. The training sessions covered specific topics, such as accessing and installing the app on personal smartphones, completing the digital data collection form, participant anonymization, image capture procedures, recruitment guidelines, informed consent, distribution of monitoring materials (rulers and markers), and instructions for resolving questions or participant withdrawal.

The external validation process of the clinical algorithms involved comparing the wound types recorded by the nurses with those automatically generated by the algorithms. This comparative analysis was conducted internally and remained inaccessible to the collaborating nurses, ensuring blinding and avoiding influence on clinical decisions.

This observational study did not involve the application of direct interventions but the use of an app for data collection, image capture, and confirmation of the processed information. While using the app, local nurses received feedback on wound size, recommendations, and alerts. It is important to highlight that the information was initially recorded by the user, later submitted to the application for analysis by the algorithms, and only then was the feedback given. The final clinical decision remained the responsibility of the collaborating nurse, regardless of the decision issued by the app, thus ensuring the person’s safety.

We tried to ensure that the same nurse always collected information from the same patient and wounds over the 4‐week follow‐up period.

An additional potential risk lay in recording information through the app, which consumed time and resources. However, we ensured that participation in the study did not interfere with other clinical activities carried out by participants; since wound monitoring is already included in their daily routines, they would only have to record the information in the app.

### 2.7. Sample Size

The sample size was determined based on the level of agreement between what was recorded by the collaborating nurse (user) and what was suggested by the algorithms, considered ideal for the kappa coefficient, set at 0.8, while values below 0.5 were considered unacceptable agreement. Furthermore, to estimate the sample size, a 95% CI, 80% statistical power, and an estimated prevalence of 50% for wounds were adopted, considering that there are different types of wounds with different prevalences. Using the formula proposed by Donner and Eliasziw [25], which is applied to determine the minimum number of observations necessary to detect acceptable agreement between evaluated observers. The sample size was set at 66 distinct wounds, including PI/PU in different clinical presentations, arterial ulcers, VLU, mixed LU, LU of unknown etiology, DFU, neuropathic DFU, ischemic DFU, and neuroischemic DFU.

### 2.8. Data Analysis

Descriptive statistics (frequencies, means, and standard deviations) were used to summarize sample characteristics and wound parameters. Concordance between algorithm‐generated classifications and nurse assessments was analyzed using Cohen’s kappa, Fleiss’s kappa, and Krippendorff’s alpha, with 95% confidence intervals. Krippendorff’s alpha was used to assess the overall level of agreement between the algorithm‐generated classifications and the reference classifications across cases in which a wound typology classification was produced; therefore, it was not applied to cases with missing information, such as absence of a photo or insufficient clinical data. A chi‐square test was applied to assess whether agreement with therapeutic recommendations exceeded a reference threshold of 70%. Statistical significance was set at *p* < 0.05.

Interpretation of concordance coefficients followed established guidelines: ≤ 0 = no agreement, 0.01–0.20 = slight, 0.21–0.40 = fair, 0.41–0.60 = moderate, 0.61–0.80 = substantial, 0.81–1.00 = almost perfect [26–28]. Analyses were conducted using IBM SPSS Statistics Version 28 (IBM Corp., New York, USA) and R Version 4.3.0 (R Foundation for Statistical Computing, Vienna, Austria).

## 3. Results

### 3.1. Sample Characteristics

The study included seven health institutions of varying types, reflecting the diversity of clinical contexts where complex wounds are managed: National Network of Integrated Continuing Care (30.6%), hospital medical wards (36.7%), nursing homes (10.2%), outpatient clinics (10.2%), community care units (10.2%), and home care (2%). Hospitalization was the most frequent care episode (85.7%).

A total of 49 patients with 56 wounds were enrolled. The mean age was 79 years (SD = 12.98; range 55–104), and 57.1% were male. Most participants had skin phototypes II or III. Clinical characteristics are summarized in Table 2. Hypertension (59.2%), diabetes mellitus (32.7%), and anemia (26.5%) were the most common comorbidities. Contributing factors to delayed healing included friction/pressure/shear forces (90.9%) and partial or total immobility (54.5%).

**TABLE 2:** Participant characteristics (*n* = 49).

Variable	*n* (%) or mean (SD)
Body mass index (kg/m^2^)	27.82 (9.9)
Tobacco use in the last 6 months (yes)	2 (4.4%)
History of previous vascular surgery (yes)	9 (20%)
Allergies (yes)	3 (5.4%)
Associated diseases (present):	
Hypertension	29 (59.2%)
Other	19 (38.8%)
Diabetes mellitus (DM)	16 (32.7%)
Anemia	13 (26.5%)
Medication (yes):	
Other	22 (44.9%)
Anticoagulants	19 (38.8%)
Factors contributing to delayed healing (present):	
Friction, pressure, or shear forces	30 (90.9%)
Partial or total immobility	18 (54.5%)
Incontinence/moisture	11 (33.3%)
Edema	9 (36%)

Abbreviations: DM = diabetes mellitus, SD = standard deviation.

### 3.2. Wound Characteristics

PI/PU were the most prevalent wound type (71.4%), followed by VLU (7.1%) and arterial ulcers (7.1%). All categories of PI/PU were represented, with categories 3 (25%) and 4 (23.2%) being most frequent. Previous ulcer history was reported in 23.2%.

Wounds were located exclusively on the trunk and lower limbs, most often in the sacrococcygeal region (19.6%), calcaneus (16%), buttock region (14.3%), trochanteric region (12.6%), and the lower third of the leg (10.7%).

In complete monitoring sessions (*n* = 109), the mean wound area was 14.33 cm^2^ (SD = 19.7), and the mean wound depth was 2.25 cm. On average, the wound bed consisted of 53.9% granulation tissue (*n* = 97), 33.9% slough (*n* = 75), and 12.1% necrotic tissue (*n* = 23). Signs of infection were identified in 27 assessments, with the most frequent being the presence of necrotic/slough tissue (*n* = 31), increased exudate (*n* = 20), and delayed healing over 2–4 weeks (*n* = 19). Wound edges were most described as macerated (*n* = 36) or rolled/thickened (*n* = 19). Maceration was also the most frequent finding in the perilesional skin (*n* = 55). Pain was generally low to moderate (mean = 3.91 on a numerical scale), most commonly intermittent and neuropathic in nature, and higher in venous and arterial ulcers.

### 3.3. Monitoring

A total of 109 complete monitoring sessions and 30 partial sessions were performed, resulting in 95 wound images. The number of complete sessions was lower than the expected 168 (estimated from the number of wounds included and the scheduled number of monitoring sessions per wound) due to wound healing, discharge, transfer, or death.

Complete monitoring generally required 10–30 min, while partial monitoring was completed in under 10 min. Monitoring times varied by wound type, with VLU and PI/PU requiring the most time (Table 3).

**TABLE 3:** Time required for complete monitoring by wound type (*n* = 96 observations).

Time (min)	DFU	PI/PU	Device‐related PU	Arterial ulcers	Mixed LU	VLU
< 10	0	23	3	1	0	2
10–20	2	32	1	5	4	2
21–30	0	7	0	3	3	1
31–40	0	2	0	0	0	1
41–50	0	1	0	0	0	2
> 50	0	1	0	0	0	0

Abbreviations: DFUs = diabetic foot ulcers, LUs = leg ulcers, PI/PU = pressure injury/pressure ulcer, VLUs = venous leg ulcers.

### 3.4. Concordance Analysis

The algorithm generated a wound typology classification in 46 of 56 cases; in the others, the lack of suggestions was due to the absence of clinical information, such as associated diseases or conditions, body mass index, history of vascular surgery, wound recurrence, presence of specific signs and symptoms, vascular assessment, and relationship of the wound with bony prominences or medical devices. We observed a fair overall level of agreement of 0.406 (CI 0.243–0.569). The point estimate is below 0.5, but the CIs include a value of 0.5, indicating that the concordance can be considered statistically equal to 0.5. Thus, within the constraints of a pilot study, these values may be considered acceptable, given the large number of categories with small subsamples. Krippendorff’s *α* (0.39, 95% CI: 0.22–0.57) and Fleiss’s *κ* (0.39, 95% CI: 0.25–0.53) yielded similar results (Table 4).

**TABLE 4:** Global concordance between nurse evaluations and algorithmic recommendations.

Statistic	Estimate	SE	95% CI
Cohen’s *κ* (unweighted mean)	0.406	0.083	0.243–0.569
Krippendorff’s *α*	0.395	0.087	0.222–0.570
Fleiss’ *κ*	0.390	0.070	0.253–0.527

By wound type, concordance was almost perfect for DFU and arterial ulcers, moderate for PI/PU, and poor for VLU and device‐related PI/PU, though estimates were limited by small subsample sizes (Table 5).

**TABLE 5:** Concordance between nurse and algorithmic assessments by wound typology (Fleiss’ *κ*).

Category	Estimate	SE	95% CI
DFU—ischemic	−0.009	0.134	−0.271–0.253
DFU	1.000	0.134	0.738–1.000
PI/PU	0.567	0.134	0.305–0.829
Device‐related PI/PU	−0.018	0.134	−0.280–0.244
Arterial ulcers	0.848	0.134	0.586–1.000
Mixed LU	0.372	0.134	0.110–0.634
VLU	−0.037	0.134	−0.299–0.225
LU—unknown etiology	0.296	0.134	0.034–0.558

Abbreviations: DFUs = diabetic foot ulcers, LUs = leg ulcers, PI/PU = pressure injury/pressure ulcer, VLUs = venous leg ulcers.

Negative Fleiss’ *κ* values should be interpreted with caution, as they likely reflect estimation uncertainty resulting from small subsamples and imbalanced category distributions rather than true disagreement.

### 3.5. Therapeutic Adherence and Alerts

Nurse adherence to therapeutic recommendations was high. In complete monitoring sessions (*n* = 109), 85.3% of recommendations were followed, *χ* ^2^(1) = 50.82, *p* < 0.001. In partial monitoring sessions (*n* = 30), adherence reached 90.3%, *χ* ^2^(1) = 19.59, *p* < 0.001.

In the clinical algorithm supporting the diagnosis of wound typology, a mean of two alerts were issued (mean 1.5; minimum 0; maximum 6) for each registered wound, with the most frequent alert being “Nutritional risk: It is recommended to perform nutritional screening and assessment in accordance with the institution’s policies.” In the clinical algorithm supporting the therapeutic plan, a mean of 4 alerts were issued (mean 3.5; minimum 0; maximum 12) for each monitoring, the most frequent being the alert “Attention: The user presents factors that contribute to delayed healing.”

Nurses rated the alert system positively, with mean scores of 4.34 (SD = 0.82) in complete monitoring sessions (*n* = 109) and 4.33 (SD = 0.97) in partial monitoring sessions (*n* = 30).

### 3.6. Final Nurse Feedback

At the end of the study, 11 nurses completed a qualitative feedback questionnaire (mean age = 34.5 years; 55% female). Most (82%) highlighted the importance of training before implementation. All agreed that the app facilitated wound monitoring, emphasizing improvements in data organization, continuity of care, and decision‐making. Reported benefits included “*a more uniform and succinct way of monitoring*” and “*better and more accurate monitoring of wound evolution*.” Regarding patient acceptance, all 11 nurses reported that they had not noticed any discomfort or distrust on the part of patients related to the use of the equipment. Challenges included the time required for initial data entry and the use of personal devices. Suggested improvements were automation of tissue characterization, refinement of therapeutic recommendations, and reducing registration time.

## 4. Discussion

This pilot study explored the feasibility of implementing clinical algorithms in an app to support decision‐making in complex wound care. Findings suggest that algorithm‐driven tools may be clinically feasible across diverse clinical settings, are generally well accepted by nurses, and show fair preliminary concordance with clinical assessments. However, successful integration into practice requires consideration not only of clinical feasibility but also of organizational and technical feasibility, as well as usability.

Our results contribute to the growing body of evidence on CDSS in wound management. Previous studies have often focused on technical validation or laboratory‐based testing [29], whereas this study evaluated performance in real‐world practice, addressing a recognized gap in the literature [18].

Nurses required 10–30 min to complete assessments with the app, which is shorter than times reported in surveys where wound care often required 15–60 min [9]. These findings suggest that algorithmic guidance may help streamline wound care processes. Nurse adherence to therapeutic recommendations was high (85%–90%), exceeding the range reported in the systematic review by Akbar et al., in which agreement with nursing decision support recommendations varied from 20% to 87% across different CDSSs [12]. Although Khong et al. also reported agreement above 90%, this referred to expert concordance with the content generated by a PI/PU CDSS, not to clinical validation in real‐world settings [30]. As such, while the present findings are broadly consistent with previous work supporting the potential of decision support tools, direct comparisons should be made with caution given the substantial differences in study design, type of system, and evaluation context.

The literature supports the potential of CDSS to enhance quality of care. For example, algorithm‐supported management of category 2–3 PI/PU improved healing outcomes compared with standard care [31]. Implementation of a CDSS in hospital settings improved documentation of preventive interventions and reduced the incidence of PI/PU [13]. Similarly, Moore et al. [9] reported that an app for wound assessment increased nurses’ confidence and reduced practice variation. Nonetheless, barriers remain, identified as concerns about costs, perceived workload, and resistance to change as factors limiting adoption [32]. Our findings of high adherence and positive feedback are encouraging but must be interpreted considering these broader challenges.

### 4.1. Implications for Clinical Practice

The integration of clinical algorithms into the app has the potential to reduce practice variation, improve diagnostic consistency, and support structured, evidence‐informed decision‐making. These tools may also enhance interdisciplinary communication, continuity of care, and person‐centered planning. By supporting standardized assessment and therapeutic planning, algorithms may help optimize nursing interventions, reduce errors, and ultimately improve quality of life for patients and families while reducing healthcare costs [9, 10, 33, 34]. However, implementation pathways and workflow integration remain to be systematically evaluated.

This pilot study suggests that integrating clinical algorithms into an app can contribute to reducing variability in wound assessment and therapeutic decision‐making. By providing structured and evidence‐based guidance, such tools help ensure that nurses follow consistent standards of practice across different care settings [35]. Standardized decision pathways may support more accurate diagnoses and therapeutic plans, minimizing errors in wound classification and promoting continuity of care.

Moreover, for nurses working in resource‐limited contexts, mobile decision‐support tools can offer just‐in‐time guidance, reinforcing safe and efficient practice even when specialist resources are not readily available [36].

Although this study was not designed to evaluate the effectiveness of the app on wound healing outcomes, the findings demonstrate its potential clinical utility in supporting diagnostic accuracy and consistency, contributing to safer and more standardized wound care practices.

### 4.2. Limitations

Despite rigorous methods, several limitations constrain interpretation. The sample size was smaller than planned, limiting statistical power and the precision of concordance estimates. Subgroup analyses by wound type were based on very small numbers, and some yielded unstable or negative kappa values. Incomplete data entry, particularly missing vascular assessments, also limited the accuracy of algorithm outputs. This issue underscores the importance of standardized and comprehensive data collection protocols. Additionally, the validation process did not involve an expert panel as a gold standard, which may have limited the ability to identify systematic misclassifications, a step recommended in CDSS validation research [12, 30]. Therapeutic recommendations were evaluated only in terms of nurse adherence, not their impact on wound healing or patient outcomes. As a result, the study cannot establish causal links between algorithm use and clinical effectiveness. Furthermore, the use of personal devices and the variable digital literacy of nurses may have introduced bias.

Moreover, the construct of *user positivity*, which was intended to reflect nurses’ subjective evaluation of the alert system, representing satisfaction and perceived usefulness on a 1–5 Likert scale, functions as a proxy for alert usability and clinical relevance. However, this measure remains only partially explored and would benefit from further validation or use of standardized usability instruments.

Data integrity was a clear limiting factor due to the absence of mandatory fields, highlighting the need for workflow redesign, mandatory field checks, and interoperability with the electronic health record.

Another limitation is the potential inter‐nurse variability, which was not systematically assessed. Although each patient was followed by the same nurse, differences in training, experience, or attitudes toward digital tools may have influenced how nurses perceived or interacted with the app. Inter‐nurse variability thus remains an unmeasured factor that may have contributed to observed discrepancies, and future research should explicitly explore patterns of agreement across different nurses [37].

Future studies should employ larger and more diverse samples with extended follow‐up periods to more robustly assess the effects of algorithm‐supported care on wound healing dynamics, complication rates, and patients’ quality of life. Randomized controlled trials, ideally complemented by multicenter pragmatic designs, are needed to establish causal relationships between algorithm use and meaningful clinical outcomes. Improving data entry standardization, combined with integration of automated features such as tissue characterization and wound measurement through advanced image analysis, may enhance usability, reduce variability, and increase diagnostic precision [38, 39]. External validation of algorithm outputs by expert panels in wound care would strengthen reliability and align results with established clinical practice guidelines [30]. Finally, future implementation studies should explore barriers and facilitators to adoption, including workload, training requirements, costs, interoperability with electronic health records, and clinician acceptance, to support scalable and sustainable integration across varied healthcare environments [39, 40].

## 5. Conclusion

This pilot study suggests that clinical algorithms can be feasibly integrated into a mobile app to support the assessment and management of complex wounds. The findings showed high nurse adherence to therapeutic recommendations, positive perceptions of the alert system, and fair preliminary concordance with clinical assessments, underscoring the potential of algorithm‐driven tools to promote standardized, evidence‐informed wound care. In addition to providing empirical feasibility data, this study offers a structured model for algorithm‐supported nursing decision‐making. Nevertheless, the findings should be interpreted cautiously given the small sample size, incomplete data, and lack of gold standard validation. Further research involving larger and more diverse samples, expert validation, and more rigorous study designs is required to confirm clinical effectiveness and support implementation in routine practice.

## Funding

This work was supported by Fundação para a Ciência e a Tecnologia, by project reference UID/04279/2025 and DOI identifier https://doi.org/10.54499/UID/04279/2025 (Centro de Investigação Interdisciplinar em Saúde).

## Conflicts of Interest

The authors declare no conflicts of interest.

## Data Availability

The data that support the findings of this study are available from the corresponding author upon reasonable request.
